# Mental health, violence and psychological coercion among female and male trafficking survivors in the greater Mekong sub-region: a cross-sectional study

**DOI:** 10.1186/s40359-018-0269-5

**Published:** 2018-12-12

**Authors:** Lisbeth Iglesias-Rios, Siobán D. Harlow, Sarah A. Burgard, Ligia Kiss, Cathy Zimmerman

**Affiliations:** 1 Department of Epidemiology, School of Public Health, University of Michigan, 1415 Washington Heights, Ann Arbor, MI 48109 USA; 2 Department of Sociology, College of Literature Science, and the Arts, University of Michigan, Ann Arbor, MI USA; 3 Gender Violence and Health Centre, Department of Global Health and Development, London School of Hygiene and Tropical Medicine, London, UK

**Keywords:** Human trafficking, Forced labor, Violence, Coercion, Anxiety, Depression, PTSD, Females, Males

## Abstract

**Background:**

Human trafficking is a pervasive global crime with important public health implications that entail fundamental human rights violations in the form of severe exploitation, violence and coercion. Sex-specific associations between types of violence or coercion and mental illness in survivors of trafficking have not been established.

**Methods:**

We conducted a cross-sectional study with 1015 female and male survivors of trafficking (adults, adolescents and children) who received post-trafficking assistance services in Cambodia, Thailand or Vietnam and had been exploited in various labor sectors. We assessed anxiety and depression with the Hopkins Symptoms Checklist (HSCL-25) and post-traumatic stress disorder (PTSD) symptoms with the Harvard Trauma Questionnaire (HTQ), and used validated questions from the World Health Organization International Study on Women’s Health and Domestic Violence to measure physical and sexual violence. Sex-specific modified Poisson regression models were estimated to obtain prevalence ratios (PRs) and their 95% confidence intervals (CI) for the association between violence (sexual, physical or both), coercion, and mental health conditions (anxiety, depression and PTSD).

**Results:**

Adjusted models indicated that for females, experiencing both physical and sexual violence, compared to not being exposed to violence, was a strong predictor of symptoms of anxiety (PR = 2.08; 95% CI: 1.64–2.64), PTSD (PR = 1.55; 95% CI: 1.37–1.74), and depression (PR = 1.57; 95% CI: 1.33–1.85). Among males, experiencing physical violence with additional threats made with weapons, compared to not being exposed to violence, was associated with PTSD (PR = 1.59; 95% CI: 1.05–2.42) after adjustment. Coercion during the trafficking experience was strongly associated with anxiety, depression, and PTSD in both females and males. For females in particular, exposure to both personal and family threats was associated with a 96% elevated prevalence of PTSD (PR = 1.96; 95% CI: 1.32–2.91) and more than doubling of the prevalence of anxiety (PR = 2.11; 95% CI: 1.57–2.83).

**Conclusions:**

The experiences of violence and coercion in female and male trafficking survivors differed and were associated with an elevated prevalence of anxiety, depression, and PTSD in both females and males. Mental health services must be an integral part of service provision, recovery and re-integration for trafficked females and males.

## Background

Modern slavery is the term that has emerged recently to encompass extreme forms of exploitation, including human trafficking, forced labor and forced marriage [1, 2]. These abuses are a pervasive global phenomena that have important implications for public health and human rights [1, 2]. Estimates suggest that 40.3 million women, men, and children are in modern slavery situations, with 24.9 million exploited as forced laborers in different economic sectors (e.g., fishing, agriculture, construction, domestic work) and 15.4 million in forced marriage conditions [2]. The Asia Pacific region accounts for the largest number of forced laborers, at 62% of the global total, where four out of every 1000 people suffer from labor exploitation [2].

 Perpetrators of trafficking often assert their control and coercion, which are well-recognized tactics related to interpersonal violence (sexual, psychological, or physical), and frequently rely on taking advantage of an individual’s particular vulnerabilities (e.g., age, sex) [3–6]. For instance, it is known that females trafficked for commercial sex and domestic work are subjected to high levels of sexual violence [4, 6]. Additionally, perpetrators may take advantage of the inherent characteristics of the particular labor sector, such as its informality or engagement of irregular migrants, to assert their power over trafficked individuals [7]. This complex asymmetrical manifestation of power, control and violence over trafficked individuals and the fact that different forms of trafficking tend to be sex specific (i.e., females are more commonly exploited for sex work, men for fishing or construction) suggests that experiences of violence (e.g., sexual, physical or both) and coercion will differ for females and males [8–11]. However, to date, there has been limited sex-specific or comparative evidence on the mental health impact of human trafficking for different forms of labor exploitation or among culturally diverse populations of females and males (including adults, adolescents, and children). Most research on human trafficking has centered on sex trafficking and females, while other forms of forced labor and trafficking of males remains understudied. This in turn has led to an underestimate of the number of females and males affected by the different forms of exploitation in most locations [12, 13].

Scholars and experts have argued that “exploitation” is at the core of the definition of human trafficking or forced labor and that “coercion” is a key feature [4, 11, 14–16]. Psychological coercion (threats or deception) and violence (physical, sexual, or both) are interconnected and can be sources of traumatic and chronic stressors [3, 4, 17]. Indeed, trafficked individuals report similar experiences of violence, coercion and corresponding psychological consequences as prisoners of war, torture survivors, survivors of concentration camps, cult members, and victims of domestic violence [3, 4, 18–24]. Perpetrators exert high levels of power and control over victims’ social, physical, psychological, sexual or economic milieu using systematic organized techniques of disempowerment and disconnection [4, 11, 14].

The substantial inequalities in power and control that are experienced by trafficked individuals are associated with higher levels of physical, psychological, and sexual violence [3, 25]. Deprived living conditions with profound restrictions on basic human needs (e.g., food, water, and shelter) during trafficking, and the fact that enslaved individuals may not be able to predict or control any aspect of their life circumstances, make trafficked individuals more susceptible to chronic disease and mental illness [11, 26–29]. Studies on human trafficking in females that used screening instruments to assess mental health have reported a very high prevalence of anxiety (48.0–97.7%), depression (52.0–100%), and post-traumatic stress disorder (PTSD, 19.5–77.0%) [26, 30–33]. Only three studies have evaluated the mental health of trafficked males [34–36]. One [34] examined symptom associations with different levels of violence, while another reported only descriptive results for 27 males [36]. The third study included only 18 trafficked men who were in contact with secondary mental health services [35], therefore the past evidence is limited.

Studies conducted with trafficked females and males suggest that there are sex-related differences in survivors’ mental health. The present study builds on earlier findings from the Study on Trafficking, Exploitation and Abuse in the Mekong Sub-region (STEAM) [34]. STEAM is a pioneering study as the first and largest health survey of trafficking survivors exploited in various labor sectors among a diverse Southeast Asian population of females and males, including children and adolescents using post-trafficking services [34]. The aim of the study was to examine the experience of violence and coercion in relation to mental health (anxiety, depression, and PTSD) of female and male trafficking survivors. To our knowledge, no studies to date have assessed the sex-specific associations between types of violence or coercion and mental illness in survivors of trafficking. Throughout the paper, the terms human trafficking and forced labor are used interchangeably.

## Methods

### Data source, study design, and study sample

This study is a cross-sectional secondary analysis using data from the STEAM. The study methodology has been published elsewhere [34]. The study sample included 1015 survivors: trafficked males, females, adolescents, and children (aged 10–17 years) who reached the country of exploitation and attended post-trafficking assistance services in Cambodia, Thailand, or Vietnam.

### Sample design

A two-stage sampling strategy was used to identify individuals using post-trafficking services. First, 15 post-trafficking support service organizations were selected across the three countries (6 services in Cambodia, 4 in Thailand, and 5 in Vietnam) based on diversity of clientele (e.g., age, sex, sector of exploitation, and country of origin), service relationship with the International Office of Migration (IOM) country teams, and agreements with government agencies (e.g., support, referral, and service arrangements). The STEAM describes individuals who received post-trafficking services, regardless of differing legal definitions of trafficking and service eligibility criteria between countries [34].

Second, a consecutive sample of individuals were invited to participate in a structured interview within 2 weeks of admission to the post-trafficking services between October 2011 and May 2013. Participants were recruited only if the locally-trained caseworker or social worker determined that their participation would not cause harm to their well-being. Individuals in the sample were identified as trafficked by the local governmental and non-governmental referral networks and post-trafficking service providers. The response rate for the baseline survey was 98%.

### Data collection

Interviews were conducted by caseworkers or social workers from the agencies providing post-trafficking services. Interviewers received an intense one-week training provided by one of the principal investigators of the STEAM (LK) in collaboration with the IOM partners in each country. Data collection and double data entry were coordinated by IOM country offices, with oversight by the London School of Hygiene and Tropical Medicine (LSHTM).

### Development of survey questionnaire and application

The survey questionnaire was based on the instrument used in a previous European study on health and sex trafficking [28] and adapted by the study team for the different study populations (various labor forms of exploitation) and the regions studied by STEAM. The interviewers also participated in adapting the questionnaire, which was pilot tested in the study settings. The survey included questions about socioeconomic background, pre-trafficking and post-trafficking exposures, living and working conditions during trafficking, violence and coercive factors, mental and physical health outcomes, and future plans and concerns. The instrument was translated into Khmer, Thai, Vietnamese, and Lao in multiple steps: professional translation from English to other languages, group translation-discussion processes with IOM counter-trafficking teams, pilot-testing, and review after back-translation into English.

### Ethics

A strict ethical and safety protocol was implemented based on the *World Health Organization (WHO) Ethical and Safety Recommendations for Interviewing Trafficked Women* [37]. Ethical approval for the study was granted by the LSHTM and by the National Ethics Committee for Health Research in Cambodia, the Hanoi School of Public Health in Vietnam, and the Ministry of Social Development and Human Security in Thailand. Core ethical guidance included measures to ensure that participation was voluntary and confidential, assurance that declining participation would not affect the provision of support services, avoidance and management of distress, and the offering of options for supported referral for health or other problems. The secondary analysis was approved by the University of Michigan Health Sciences and Behavioral Sciences Institutional Review Board, eResearch ID: HUM00097096.

### Specific measures

#### Anxiety, depression, and post-traumatic stress disorder symptoms measures

Anxiety and depression symptoms in the past week were measured by the Hopkins Symptom Checklist**-**25, a symptom inventory [38]. It consists of 25 items: 10 for anxiety symptoms and 15 for depression symptoms. The scale for each item includes four categories of response (“Not at all,” “A little,” “Quite a bit,” and “Extremely,” rated 1 to 4, respectively). The anxiety score was calculated as the average of the anxiety items, while the depression score was the average of the depression items. The depression score has been correlated with major depression as defined by the Diagnostic and Statistical Manual of the American Psychiatric Association*,* 4th edition (DSM-IV) in several populations [39]. A cutoff of 1.625 instead of the established value of 1.75 was used to identify symptoms of depression, as item 12 in the questionnaire (i.e., loss of sexual interest or pleasure) was excluded, given the nature of the study population [34]. For anxiety, a cutoff of 1.75 indicated symptoms of anxiety, based on previous research on individuals using post-trafficking services and on studies of Cambodian, Laotian, and Vietnamese refugees with whom this instrument has been validated [31, 36, 40, 41].

PTSD symptoms in the past week were measured using the Harvard Trauma Questionnaire (HTQ) part IV, which includes 27 trauma symptoms [42]. The first 16 items were derived from the DSM-IV criteria for PTSD and assessed the presence of the main PTSD symptom clusters: intrusive experiencing, avoidance behaviors, hypervigilance, and emotional numbing [39]. The remaining items were developed by the Harvard Program in Refugee Trauma. These PTSD symptom items focus on the impact that the traumatic experiences may have had on the subject’s perception of his or her daily life (e.g., having difficulty dealing with new situations) [42–44]. Each question has four response categories: “Not at all,” “A little,” “Quite a bit,” and “Extremely,” rated 1 to 4, respectively. A total score was calculated by averaging the 27 items. A cutoff of 2.0 was used to assess symptoms of PTSD based on previous research on trafficked individuals accessing post-trafficking services [30, 32]. Although the HTQ has not been validated with the study population, it has been used in cross-cultural settings and among Southeast Asian populations (e.g., Cambodians) exposed to trauma [40, 45, 46]. This instrument has shown high sensitivity for identifying persons with PTSD when diagnosed by experienced psychiatrists in a clinical setting and according to DSM criteria [40]. The HTQ has high reliability [47] and internal consistency [47, 48] and test-retest reliability ranging from 0.89 to 0.92 [48, 49].

#### Violence and coercion measures

To assess physical and sexual violence, standardized and validated questions from the World Health Organization (WHO) International Study on Women’s Health and Domestic Violence were used [13]. These questions describe acts of physical and sexual violence commonly reported by trafficked individuals in post-trafficking services and shelters [4, 30, 34, 36]. For females, we created a three category indicator of: “no violence”, “physical violence only”, “sexual violence only”, and “physical and sexual violence.” “Physical violence only” indicated the experience of any violent acts such as: being kicked, dragged, or beaten up; being tied or chained, choked, or burned; having a dog released to bite or scratch; being threatened with a weapon, cut with a knife, or being shot at, experiencing punches, slaps, and hits, but no experience of sexual violence. “Sexual violence only” was defined by a positive response to one item asking whether the respondent was forced to have sex, but no experience of physical violence, and lastly, those in the “physical and sexual violence” category were exposed to both types. These categorizations were based on previous research with survivors of trafficking [26, 28, 34]. For males, violence (yes/no) was measured with the variable “physical violence with additional threats made with a gun, knife, or other weapon” as only six males reported sexual violence. This variable included all the acts of physical violence listed above with additional threats made with weapons. We also identified in descriptive analysis that the experience of violence in males was for the most part defined by this type of physical violence.

Two additional questions were used to assess coercion during the trafficked period for females and males: (a) “While you were in this situation, did anyone threaten to hurt you?” (yes or no) and (b) “During this time did anyone threaten to hurt your family or someone you care about?” (yes or no). These questions assess threats commonly made by traffickers that are considered hallmarks of the trafficking experience and are frequently used in studies of interpersonal violence [14, 28, 34, 50].

#### Covariates

Covariates in this analysis were theory-driven and based on prior analyses of the STEAM [11, 32, 34, 51], and included age (10–17, 18–25, and 26 or above), country of exploitation and trafficking (Thailand, China, or Other [Cambodia, Malaysia, Vietnam, Indonesia, Mauritius, South Africa, and Russia]), and time in trafficking (1–12 and 13 or more months). Participants were asked which trafficking sector they were exploited in most recently. The grouping of sectors of exploitation was based on similarity of occupational exposures and risks, balanced with the need to group sectors together due to low counts in particular occupations. Sectors for females and males were grouped together as sex work, forced marriage, entertainment, and dancing (sex and entertainment industry); domestic work, cleaning, restaurant work, and begging (hospitality industry and begging); construction and factory work (manufacturing industry); and livestock, meat packing and preparation, agriculture, or fishing (animal and agriculture industry). For males, we further collapsed the sex and entertainment industry with the hospitality industry and begging sectors to be able to make meaningful comparisons, since there were few individuals in those sectors. Groupings of sectors of exploitation were also based on previous research that indicates that some of these labor sectors might share similar levels of violence [32, 34, 52].

#### Statistical analyses

Because of important differences in the distribution of violence, coercion and trafficking-related exposures, the analyses were stratified by sex. We calculated frequencies and conducted bivariate analyses with cross tabulations using Rao-Scott chi-square tests to account for the clustered structure of the data (i.e., post-trafficking service organizations) and assess associations between violence, coercion (threats) and covariates with anxiety, depression, and PTSD symptoms [53]. Sex-specific unadjusted and adjusted modified Poisson regression models were conducted to estimate prevalence ratios (PRs) and their 95% confidence intervals (CIs) for the associations between violence and coercion with anxiety, depression, and PTSD [54].

Generalized estimation equations (GEEs) with an extension of the sandwich variance estimator were used to calculate a robust variance estimation that considers the level of correlation of observations within a cluster and produces standard errors of the estimates accordingly [55]. This statistical approach was chosen because it is considered to be a direct and less-biased approach to estimating the PRs. This method corrects standard errors, considers clustered data [54, 56, 57], and it is robust to the specification of the working correlation structure chosen [58].

To determine the best fit of the model and the working correlation structure, we used the quasi-likelihood under the independence model criterion (QIC) statistic, which is robust to the selection of correlation structure [59]. We chose an exchangeable correlation structure that assumes that all pairs of observations are correlated within a cluster. We fit separate and sex-specific binary modified Poisson regression models for each of the outcome variables (anxiety, depression, and PTSD). We fit a crude model for females and males with the previously specified demographic covariates only and separate crude and adjusted models for violence (model 1) and coercion (model 2) with each of the mental health outcomes. Given the low number of missing data (e.g., one female and one male were missing for anxiety, depression, and PTSD) we allowed for listwise deletion in all analytical models. All tests were two-tailed and analyses were performed using SAS version 9.4 (SAS Institute, Inc., Cary, NC). PROC SURVEYFREQ with a cluster and *chisq* statement were used for the descriptive analysis. PROC GENMOD was used with the robust variance estimator provided by the REPEATED statement with a cluster identifier that uses the method of GEE to estimate the model and give a proper estimate of the standard error of the PRs while accounting for clustering in the data.

## Results

### Sample characteristics of females and males

Table 1 presents descriptive characteristics of the study population stratified by sex. A total of 569 (56.1%) females and 446 (43.9%) males participated in the survey. The mean age (±SD) of the study population was 22.8 years old ± 8.4 years. Almost half of the females were children and adolescents (< 18 years of age, 49.4%), from Vietnam (41.8%). Males were mainly 18–25 years old (45.1%), or older than 25 years old (40.8%) and most frequently were from Cambodia (57.6%). More than half of the population of females was exploited in Thailand (54.1%) and more than a third in China (39.2%). In contrast, 31.4% of males were trafficked in Thailand, 24.0% were trafficked in China.

**Table 1:** Sociodemographic characteristics of trafficking survivors by sex: The Study on Trafficking, Exploitation and Abuse in the Mekong Sub-region (STEAM), n = 1015

	Females	Males	
	n (%)	n (%)	*p*-value
Total	569 (56.1)	446 (43.9)	
Age			0.03
10–17	281 (49.4)	63 (14.1)	
18–25	197 (34.6)	201 (45.1)	
26 or above	91 (16.0)	182 (40.8)	
Country of origin			0.0001
Cambodia	49 (8.6)	257 (57.6)	
Vietnam	238 (41.8)	106 (23.8)	
Other^a^	282 (49.6)	83 (18.6)	
Country of exploitation			0.001
China	223 (39.2)	107 (24.0)	
Thailand	308 (54.1)	140 (31.4)	
Other^b^	38 (6.7)	199 (44.6)	
Sector of exploitation			0.001^d^
Sex work, forced marriage, entertainment, and dancing^c^	410 (72.1)	46 (10.3)	0.006^e^
Domestic work, cleaning, restaurant work, begging, and other	66 (11.6)		
Construction and factory work	54 (9.5)	101 (22.7)	
Livestock, agriculture, fishing	39 (6.9)	299 (67.0)	
Time in trafficking situation (months)^f^			0.002
1–12	78 (14.5)	24 (5.7)	
13 or more	460 (85.5)	401 (94.4)	
Violence during trafficking^g^			0.004^h^
No violence	295 (52.1)	219 (49.3)	
Physical violence	73 (12.9)	218 (49.2)	
Sexual violence	89 (15.7)	0 (0)	
Both physical and sexual violence	109 (19.3)	6 (1.4)	
Receiving threats during trafficking			0.0001
None	345 (60.6)	192 (43.1)	
Personal threats	141 (24.8)	206 (46.2)	
Both, personal and family threats	83 (14.6)	48 (10.8)	
Physical violence with threats made with a gun, knife, or other weapon during trafficking^i^			0.20
Yes	182 (32.2)	225 (50.5)	
No	384 (67.8)	221 (49.6)	

^a^Other country: Cambodia, Laos, Burma, Thailand, and Vietnam

^b^Other country: Cambodia, Malaysia, Vietnam, Indonesia, Mauritius, South Africa, and Russia

^c^43.9% and 25.8% of females in this sector of exploitation were exploited in Thailand and China, respectively

^d^*p*-value for females

^e^*p*-value for males. For males, we collapsed together the sex work, forced marriage, entertainment, dancing, domestic work, cleaning, restaurant work, begging, and other due to small sample size across those sectors (*n* = 46)

^f^31 females and 21 males missing

^g^3 females and 3 males missing

^h^*p*-value for females

^i^3 females missing

Most females were trafficked for sex work, forced marriage, entertainment, and dancing (72.1%) in Thailand (43.9%) and China (25.8%). In contrast, males were exploited mainly in sectors related to livestock (including meat preparation), agriculture, and fishing (67.0%) and construction and factory work (23.0%) in Thailand and various other countries (Cambodia, Malaysia, Vietnam, Indonesia, Mauritius, South Africa, and Russia). The vast majority of individuals were trafficked for more than 1 year irrespective of sex: 85.5% of females and 94.4% of males.

Approximately half of the participants, 52.1% of females and 49.3% of males, reported no experience of violence. Males experienced more episodes of physical violence only (49.2%), while for females, violence involved physical (12.9%) or sexual violence (15.7%) alone, or both physical and sexual violence (19.3%). Receiving personal threats was almost twice as common for males (46.2%) as for females (24.8%). In contrast, experiencing both personal and family threats was slightly more common for females (14.6%) than for males (10.8%). Half of the males (50.5%) and a third of females (32.2%) were subjected to physically violent acts that involved additional threats made with a gun, knife, or other weapon.

Table 2 presents information on the prevalence of anxiety, depression, and PTSD for females and males. The prevalence of depression was higher in women but present in more than half of the entire study population: 64.3% of females and 57.3% of males. PTSD was reported by about two-fifths of males (41.8%) compared to about a third of females (34.5%). Anxiety was experienced by more than two fifths in both females (40.5%) and males (45.8%).

**Table 2:** Prevalence of mental health symptoms among trafficked survivors by sex, n = 1015

	Females	Males	*p*-value
	n (%)	n (%)	
Anxiety^a^			0.08
Yes	230 (40.5)	204 (45.8)	
No	338 (59.5)	241 (54.2)	
Depression^b^			0.02
Yes	365 (64.3)	255 (57.3)	
No	203 (35.7)	190 (42.7)	
Post-traumatic stress disorder (PTSD)^c^			0.02
Yes	196 (34.5)	186 (41.8)	
No	372 (65.5)	259 (58.2)	

^a^^,^^b^^,^^c^1 female & 1 male missing for anxiety, depression, and PTSD

### Modified Poisson regression models in females

Results from the crude prevalence ratios of demographic characteristics and anxiety, PTSD, and depression are presented in Table 3.

**Table 3:** Crude prevalence ratios of demographic characteristics and anxiety, post-traumatic stress disorder, and depression in trafficked females, n = 569

	Anxiety		PTSD^a^		Depression	
	PR^b^	95% CI^c^	PR	95% CI	PR	95% CI
Age						
10–17 (reference)	1.0		1.0		1.0	
18–25	1.26	1.12–1.42^***^	1.12	0.96–1.30	1.09	0.97–1.22
26 or above	1.26	0.93–1.70	1.15	0.86–1.53	1.02	0.85–1.24
Country of exploitation						
Thailand (reference)	1.0		1.0		1.0	
China	1.34	0.87–2.07	1.57	0.81–3.01	1.15	1.01–1.32^*^
Other^d^	1.76	1.14–2.73^**^	1.80	0.89–3.63	1.05	0.56–1.94
Sector of exploitation						
Sex work, forced marriage, entertainment, dancing (reference)	1.0		1.0		1.0	
Domestic work, cleaner, restaurant work, begging, and other	1.34	1.06–1.70^**^	1.49	1.05–2.11^*^	1.12	0.89–1.42
Construction and factory work	1.50	1.12–2.03^**^	1.47	1.05–2.05^*^	1.12	0.78–1.60
Livestock, agriculture, and fishing	1.17	0.81–1.69	1.07	0.91–1.26	0.88	0.65–1.19
Time in trafficking situation (months)						
1–12 (reference)	1.0		1.0		1.0	
13 or more	1.05	0.63–1.74	1.38	0.87–2.20	1.22	0.84–1.77

^*^p ≤ 0.05

^**^p ≤ 0.01

^***^p ≤ 0.001

^a^PTSD Posttraumatic stress disorder

^b^PR Prevalence ratio

^c^95% Confidence interval

^d^Other country of exploitation included: Cambodia, Malaysia, Vietnam, Indonesia, Mauritius, South Africa, and Russia

Compared to children and adolescents (10–17 years old), the prevalence of anxiety for younger females (18–25 years old) was 26% (PR = 1.26; 95% CI: 1.12–1.42) higher. For young and older adults the prevalence of PTSD and depression was slightly elevated but it was not statistically signifcant when compared to minors. Females exploited in China had a 15% higher prevalence of depression (PR = 1.15; 95% CI: 1.01–1.32) compared to females trafficked in Thailand.

Females 1doing domestic work, cleaning, restaurant work, begging, and doing other forced labor had a 34% (PR = 1.34; 95% CI: 1.06–1.70) higher prevalence of anxiety and almost a 50% elevated prevalence of PTSD (PR = 1.49; 95% CI: 1.05–2.11) compared with those in sex work, forced marriage, entertainment or dancing. Similarly, women and girls exploited in construction and factory work had a 50% (PR = 1.50; 95% CI: 1.12–2.03) greater prevalence of anxiety and a 47% elevated prevalence of PTSD (PR = 1.47; 95% CI: 1.05–2.05). Time in trafficking was not statistically associated with any of the three mental health outcomes.

#### Crude and adjusted prevalence ratios of the association of violence and coercion (threats) with anxiety, PTSD, and depression in trafficked females

Table 4 shows the results from the crude and adjusted modified Poisson regression models for violence, coercion, and mental health outcomes in females. Multivariable models were adjusted for age, country of exploitation, sector of exploitation, and time in trafficking.

**Table 4:** Crude and adjusted prevalence ratios of the association of violence and coercion (threats) with anxiety, post-traumatic stress disorder, and depression in trafficked females, n = 569

	Unadjusted model		Adjusted Model 1^a^		Adjusted Model 2^b^	
	PR^c^	95% CI^d^	PR	95% CI	PR	95% CI
Anxiety						
Violence						
No violence (reference)	1.0		1.0			
Physical violence only	1.31	1.06–1.62^**^	1.18	0.93–1.49		
Sexual violence only	0.90	0.59–1.39	0.94	0.48–1.83		
Both physical and sexual violence	1.68	1.37–2.07^***^	2.08	1.64–2.64^***^		
Receiving threats during trafficking						
None (reference)	1.0				1.0	
Personal threats	1.93	1.54–2.41^***^			1.93	1.55–2.42^***^
Both personal and family threats	2.10	1.58–2.79^***^			2.11	1.57–2.83^***^
Post-Traumatic Stress Disorder						
Violence						
No violence (reference)	1.0		1.0			
Physical violence only	1.32	1.16–1.50^***^	1.15	0.89–1.48		
Sexual violence only	0.80	0.60–1.07	0.84	0.52–1.34		
Physical and sexual violence	1.33	1.18–1.51^***^	1.55	1.37–1.74^***^		
Receiving threats during trafficking						
None (reference)	1.0				1.0	
Personal threats	1.49	1.16–1.92^***^			1.44	1.06–1.96^*^
Both personal and family threats	1.95	1.35–2.82^***^			1.96	1.32–2.91^***^
Depression						
Violence						
No violence (reference)	1.0		1.0			
Physical violence only	1.17	0.97–1.40	1.12	0.96–1.32		
Sexual violence only	0.96	0.79–1.16	1.02	0.85–1.22		
Both physical and sexual violence	1.44	1.23–1.68^***^	1.57	1.33–1.85^***^		
Receiving threats during trafficking						
None (reference)	1.0				1.0	
Personal threats	1.46	1.18–1.81^***^			1.46	1.20–1.78^***^
Both personal and family threats	1.51	1.21–1.87^***^			1.42	1.25–1.63^***^

Models for anxiety, depression, and post-traumatic stress disorder symptoms were run separately but they were adjusted for the same variables

^*^p ≤ 0.05

^**^p ≤ 0.01

^***^p ≤ 0.001

^a^Adjusted Model 1 (violence): age, country of exploitation, sector of exploitation, time in trafficking

^b^Adjusted Model 2 (coercion or threats): age, country of exploitation, sector of exploitation, time in trafficking

^c^PR Prevalence ratio

^d^95% Confidence interval

#### Anxiety in females

Females exposed to both physical and sexual violence had a 68% greater prevalence of anxiety (PR = 1.68; 95% CI: 1.37–2.07), compared to those who did not report experiencing violence. After adjustment, the prevalence was elevated such that females exposed to both physical and sexual violence had a twofold higher (PR = 2.08; 95% CI: 1.64–2.64) prevalence of anxiety. Women and girls who suffered from physical violence alone had a 30% elevated prevalence of anxiety (PR = 1.31; 95% CI: 1.06–1.62) compared to females without violence; however, after adjustment, the estimate was reduced and they did not differ significantly from those not reporting violence. Sexual violence alone was not statistically associated with anxiety in the crude or adjusted models.

After adjustment, females who received personal threats during trafficking had a 93% (PR = 1.93; 95% CI: 1.55–2.42) greater prevalence of anxiety compared to those without threats, while the prevalence of anxiety more than doubled among those who experienced both personal and family threats (PR = 2.11; 95% CI: 1.57–2.83).

#### PTSD in females

Women and girls exposed to physical violence only, or both physical and sexual violence, had more than a 30% elevated prevalence of PTSD in the crude model. After adjustment, females exposed to both physical and sexual violence had almost a 50% higher prevalence of PTSD (PR = 1.55; 95% CI: 1.37–1.74) compared to those who did not report violence. Neither form of violence alone (physical or sexual) was statistically associated with PTSD after adjustment.

Women and girls who experienced personal threats had a 49% (PR = 1.49; 95% CI: 1.16–1.92) elevated prevalence of PTSD while for those who received both personal and family threats the prevalence for PTSD almost doubled (PR = 1.95; 95% CI: 1.35–2.82), compared to females without threats in the crude model. After adjustment, the prevalence ratio for personal threats was slightly reduced and remained statistically significant (PR = 1.44; 95% CI: 1.06–1.96). Females exposed to both personal and family threats, had a 96% greater prevalence of PTSD (PR = 1.96; 95% CI: 1.32-2.91) that remained statistically significant after adjustment.

#### Depression in females

After adjustment, females who suffered from both physical and sexual violence had a 57% (PR = 1.57; 95% CI: 1.33–1.85) higher prevalence for depression when compared to those not experiencing violence. Physical and sexual violence alone were not statistically associated with depression in crude and adjusted models. However, experiencing threats remained significantly associated after adjustment. Females who experienced personal threats had a 46% (PR = 1.46; 95% CI: 1.20–1.78) elevated prevalence for depression. Similarly, after adjustment of covariates, women and girls with both personal and family threats had a 42% (PR = 1.42; 95% CI: 1.25–1.63) higher prevalence of symptoms of depression when compared to females without threats.

### Modified Poisson regression models in males

Table 5 shows the crude prevalence ratios of demographic characteristics and anxiety, PTSD, and depression in males. Age was not significantly associated with anxiety and PTSD. However, compared to children and adolescents, older adults had a 43% (PR = 1.43;95% CI: 1.08-1.90) greater prevalence of depression. Males exploited in China and other countries had a threefold (PR = 3.63; 95% CI: 2.58–5.12) and a twofold (PR = 2.89; 95% CI: 2.04–4.11) higher prevalence of PTSD, respectively, compared to those trafficked in Thailand. The prevalence of anxiety among men and boys trafficked for 13 or more months more than doubled (PR = 2.31; 95% CI: 1.28-4.16) compared to those trafficked for 1–12 months. However, there were no statistically significant differences between the length of time in trafficking and PTSD or depression among males.

**Table 5:** Crude prevalence ratios of demographic characteristics and anxiety, post-traumatic stress disorder, and depression in trafficked males, n = 446

	Anxiety		PTSD^a^		Depression	
	PR^b^	95% CI^c^	PR	95% CI	PR	95% CI
Age						
10–17 (reference)	1.0		1.0		1.0	
18–25	1.14	0.64–2.06	1.79	0.76–4.20	1.41	0.95–2.09
26 or above	1.06	0.64–1.76	1.64	0.91–2.96	1.43	1.08–1.90**
Country of exploitation						
Thailand (reference)	1.0		1.0		1.0	
China	1.33	0.68–2.59	3.63	2.58–5.12***	1.60	0.91–2.82
Other^d^	0.92	0.70–1.22	2.89	2.04–4.11***	0.95	0.84–1.08
Sector of exploitation						
Forced marriage, entertainment, dancing, domestic work, cleaner, restaurant work, begging, and other (reference)	1.0		1.0		1.0	
Animal, farming, agriculture, and fishing	1.03	0.58–1.81	1.23	0.64–2.34	1.37	0.81–2.32
Construction and factory work	1.40	0.76–2.57	1.27	0.85–1.89	1.34	0.94–1.91
Time in trafficking situation (months)						
1–12 (reference)	1.0		1.0		1.0	
13 or more	2.31	1.28–4.16**	1.22	0.48–3.11	1.02	0.60–1.74

^*^p ≤ 0.05

^**^p ≤ 0.01

^***^p ≤ 0.001

^a^PTSD Post-traumatic stress disorder

^b^PR Prevalence ratio

^c^95% Confidence interval

^d^Other country of exploitation included Cambodia, Malaysia, Vietnam, Indonesia, Mauritius, South Africa, and Russia

#### Crude and adjusted prevalence ratios of the association of violence and coercion (threats) with anxiety, PTSD, and depression in trafficked males

Results for the crude and adjusted modified Poisson regression models for violence and coercion with anxiety, PTSD, and depression in males are presented in Table 6. Multivariable models were adjusted for age, country of exploitation, sector of exploitation, and time in trafficking.

**Table 6:** Crude and adjusted prevalence ratios of the association of violence and coercion (threats) with anxiety, post-traumatic stress disorder, and depression in trafficked males, n = 446

	Unadjusted Model		Adjusted Model 1^a^		Adjusted Model 2^b^	
	PR^c^	95% CI^d^	PR	95% CI	PR	95% CI
Anxiety						
Violence						
No violence (reference)	1.0		1.0			
Physical violence additional with threats made with weapons	1.49	1.00–2.19^*^	1.41	0.86–2.31		
Receiving threats during trafficking						
None (reference)	1.0				1.0	
Personal threats	1.36	1.14–1.62^***^			1.33	1.08–1.64^**^
Both personal and family threats	1.64	1.40–1.92^***^			1.68	1.43–1.97^***^
Post-Traumatic Stress Disorder						
Violence						
No violence (reference)	1.0		1.0			
Physical violence with additional threats made with weapons	1.60	0.92–2.78	1.59	1.05–2.42^*^		
Receiving threats during trafficking						
None (reference)	1.0				1.0	
Personal threats	1.80	1.39–2.35^***^			1.75	1.51–2.03^***^
Both personal and family threats	1.92	1.65–2.24^***^			1.62	1.31–2.00^***^
Depression						
Violence						
No violence (reference)	1.0		1.0			
Physical violence with additional threats made with weapons	1.39	0.83–2.33	1.47	0.91–2.36		
Receiving threats during trafficking						
None (reference)	1.0				1.0	
Personal threats	1.39	1.28–1.50^***^			1.46	1.35–1.57^***^
Both personal and family threats	1.42	1.24–1.62^***^			1.33	1.11–1.58^***^

Models for anxiety, depression, and post-traumatic stress disorder were run separately but they were adjusted for the same variables

^*^p ≤ 0.05

^**^p ≤ 0.01

^***^p ≤ 0.001

^a^Adjusted Model 1 (violence): age, country of exploitation, sector of exploitation, time in trafficking

^b^Adjusted Model 2 (coercion or threats): age, country of exploitation, sector of exploitation, time in trafficking

^c^PR Prevalence ratio

^d^95% Confidence interval

#### Anxiety in males

The prevalence of anxiety decreased slightly and did not differ after adjustment among those who suffered from physical violence with additional threats made with a gun, knife, or other weapon compared to those not experiencing violence. Similar to the crude model and after adjustment, the prevalence of anxiety among males was more than 30% (PR = 1.33; 95% CI: 1.08–1.64) and almost 70% (PR = 1.68; 95% CI: 1.43–1.97) higher among those who experienced personal threats and those who had experienced both personal and family threats, respectively, compared to those who had not.

#### PTSD in males

Males subjected to physical violence with additional threats made with weapons had almost a 60% higher prevalence of PTSD (PR = 1.59; 95% CI: 1.05–2.42) when compared to males without violence after adjustment. Men and boys who received personal threats, and those receiving personal and family threats, had a 75% (PR = 1.75; 95% CI: 1.51–2.03) and 62% (PR = 1.62; 95% CI: 1.31–2.00) elevated prevalence for PTSD, respectively, when compared to males without threats and after adjustment.

#### Depression in males

The prevalence of depression and physical violence with additional threats made with a gun, knife, or other weapon was elevated but did not differ significantly in crude and adjusted models. After adjustment, males subjected to personal threats and both personal and family threats had a 46% (PR = 1.46; 95% CI: 1.35–1.57) and 33% (PR = 1.33; 95% CI: 1.11–1.58) greater prevalence for depression, respectively compared to those who were not threatened.

## Discussion

The study advances knowledge about the mental health consequences of various forms of violence and coercion during trafficking among female and male survivors who were exploited in different labor sectors. We found that violence and coercion (receiving personal threats or both personal and family threats) are both independently associated with poor mental health and differed between females and males. For females, experiencing both physical and sexual violence was a strong predictor of symptoms of anxiety, PTSD, and depression, while for males, physical violence with additional threats made with weapons was strongly associated with symptoms of PTSD.

Another key finding in the study was that acts of coercion (personal and both personal and family threats) during the trafficking experience proved to be consistently and strongly associated with anxiety, depression, and PTSD symptoms in both females and males. Coercion in females was particularly strongly related to anxiety and PTSD among those receiving both personal and family threats. This finding on the influence of threats is consistent with previous research on trafficked women [28]. Our results highlight the different coercive experiences between females and males, and indicate that coercion can be as harmful to mental health as any form of physical violence.

Overall, the study results are consistent with past studies of gender-based violence and health trafficking research that report sexual and physical violence to be salient features of the trafficking experience among females [4, 12, 28, 30, 32, 34]. Previous research on human trafficking, gender-based violence and interpersonal violence indicates that experiencing more than one type of abuse (e.g., physical, sexual, and psychological or emotional) increases the probability of having anxiety, depression, or PTSD symptoms as well as the severity of those symptoms [34, 59–62]. Likewise, our results are in accordance with interpersonal violence (IPV) research that reports that psychological coercion for both men and women is strongly associated with an increased risk of symptoms of depression and PTSD [63–65].

In sum, violence and coercion for many trafficked individuals is a central element of the trafficking experience [4, 34, 66], and it was a salient feature in the present study. The systematic implementation of coercive tactics used by perpetrators reinforces control, depletes individual psychological resources and ultimately contributes to poor mental health [4, 6]. Sexual and physical violence are powerful methods to terrorize, dominate, and humiliate the enslaved individual, but sexual violence in particular is intentionally meant to produce psychological trauma [4, 15, 66]. We found that experiencing both sexual and physical violence was strongly associated with poor mental health in women and girls.

Beyond the trauma, sexual violence is also associated with an increased risk of human immunodeficiency virus (HIV) and other sexually transmitted diseases that can exacerbate physical and mental health problems [67]. Therefore, experiencing both types of violence, sexual and physical, may be an added burden to the harm inflicted on the mental health of female trafficking survivors.

Physical violence with additional threats made with weapons was an important risk factor for PTSD for males in this population. Threats with weapons are another common method to exert psychological coercion, but this is the first study to document this impact in a population of male survivors of trafficking [4, 68]. However, our finding is consistent with the relatively scant research about the nonfatal use of weapons in the context of interpersonal violence [69, 70]. Threats with weapons allow the aggressor to assert dominance in interpersonal relationships because they convey a pernicious threat, elicit compliance, and create extreme fear and intimidation, all of which are the hallmarks for coercive control [69, 70]. Psychological coercive control, a key feature of human trafficking, is known to have deleterious effects on mental health as it involves terror, fear, isolation, and helplessness which consequently affects an individual’s self-efficacy and autonomy [3, 6, 64].

Although the female burden of suffering both physical and sexual violence was expected and documented in previous research [26–29, 34, 71], the elevated prevalence for anxiety, depression, and PTSD in this population is of considerable concern. Similar elevated prevalence of depression and PTSD had been observed among populations exposed to mass conflict and displacement in which torture and trauma emerged as the strongest risk factor for PTSD and depression [72]. Overall, these findings are consistent with previous research that highlights the need to amplify our understanding of the diversity and complexity of what constitutes human trafficking and how it is carried out globally in diverse countries and through many labor sectors for females and males [34, 73].

An important limitation of this analysis is the cross-sectional design; hence causality and the temporal relationship between violence, threats, and mental health symptoms (anxiety, PTSD, and depression) cannot be established. Individuals with poor mental health could be more vulnerable to being trafficked or have a history of violence. However, previous studies on human trafficking, violence and women’s health suggest that mental health problems are more likely to be the result of abuse rather than its precursor [3, 26, 67, 74]. Assessment of mental health symptoms was conducted with screening instruments with robust psychometric properties. These instruments are commonly used in general populations in some of the study countries and in post-trafficking services. However, these instruments are not gold-standard diagnostic tools to clinically assess these disorders, therefore overestimation of the prevalence ratio of mental health outcomes is possible. It is also plausible that trafficked individuals under the harshest forms of slavery and exploitation are the ones with worse mental health and may be less likely to be reached in post-trafficking services. In this case, our estimates of the prevalence ratio will be underestimates. Nonetheless, the direction and magnitude of the associations observed are consistent with similar previous studies [26, 30, 32, 34].

The study population represents a sample of survivors of trafficking using post-trafficking services. Service eligibility and screening processes for referral to post-trafficking services may vary between countries as human trafficking is often defined according to the legal framework of the country. Therefore, the results of the study may not be generalizable to the broader population of trafficked individuals. However, this population is likely to be representative of survivors of trafficking receiving post-trafficking services in similar forced labor conditions in the countries in question, as violence and coercion are known to be core components of any human trafficking situation.

Another plausible limitation is underreporting of sexual violence in males. Overall, research on sexual abuse and sexual assault of men and boys in all settings is scarce [75, 76]. Previous studies indicate that the perpetration of sexual violence in males is more common in boys than in adult males [77–79]. Children, in particular, rarely disclose sexual abuse after the event, and disclosure tends to be a process rather than a single question or interview [76]. Male adults are more likely to be sexually assaulted by multiple assailants and to have weapons used against them [80, 81]. Sexual abuse and violence against trafficked males needs further research as well as consideration of the role that culture plays in sexual violence.

The study has important clinical, public health, and policy implications. The elevated prevalence of anxiety, depression, and PTSD in this sample of survivors indicates that addressing their mental health needs is complex and requires a coordinated, culturally sensitive, holistic, and multi-tiered system approach. Care should include immediate and continuous services (e.g., mental and physical health, social services, safety, housing, legal counsel, economic aid, and community and societal reintegration), which should be provided regardless of an individual’s country of origin, legal status, or participation in legal proceedings against traffickers. Building a network of services prepared to address the complex needs that survivors face after being trafficked is needed [7, 82].

A fundamental element of care for trafficked individuals should involve a collaborative approach and a safe space that prioritizes empowerment of survivors. Service providers need to respect survivors’ perspectives, and acknowledge their rights. Given the diverse demographic background and traumatic experiences endured by trafficked individuals, it is important that service providers be well trained with strong multicultural competencies and knowledge of the various forms of exploitation, abuse, and violence as well as ways to screen for potential cases of human trafficking.

Poor mental health and exposure to traumatic events that involve violence can have long lasting health effects, this is particularly concerning for children and adolescents. Yet, to date, the efficacy of psychological treatments for survivors of human trafficking in Southeast Asia has not been investigated. Western treatments may not fully capture the complexity of the psychological responses that arise from individuals who have experienced human rights violations [83]. However, evidence -based treatments, such as Narrative Exposure Therapy (NET) or Trauma-Focused Cognitive-Behavioral Therapy (TF-CBT), have been used with female survivors of sex trafficking and refugees [84–86]. NET was originally developed for multiple traumatized victims of organized violence in resource-poor settings, where it could be delivered by trained non-professionals [84]. A modified version of TF-CBT has been developed for children exposed to multiple complex traumas, and it is commonly used in children and adolescents with PTSD [87]. Systematic evaluation on the implementation, delivery, and outcomes of mental health interventions is essential for treatment programs, stakeholders and policy makers to improve care and bring accountability to the care of trafficking survivors.

## Conclusions

In conclusion, it is important for policy makers and stakeholders to consider the complex and severe effects that violence and coercion inflict on the mental health of survivors of trafficking. We observed differences in the experiences of violence and coercion in female and male trafficking survivors. We found a substantially elevated prevalence of anxiety (≈ 40.0%), depression (> 50%), and PTSD (> 35%) among survivors of trafficking compared to estimates of the general population in Cambodia (≈ 3.2–3.4%), Thailand (≈ 3.5–4.4%), and Vietnam (≈2.2–4.0%), respectively [88]. Our findings highlight the importance of mental health treatment as an integral part of service provision, recovery and re-integration for female and male survivors. Strengthening mechanisms to protect survivors of trafficking and family members susceptible to retaliation and coercion from traffickers is critical. Further research on the development and implementation of evidence based mental health treatments for survivors of trafficking is warranted. Public health interventions and epidemiological approaches could be valuable to furthering understanding of human slavery within a health equity framework to strengthen individuals’ and communities’ capacities to prevent and address forced labor globally.

## Abbreviations

CI: Confidence intervals
DSM: Diagnostic and Statistical Manual of the American Psychiatric Association
GEE: Generalized estimation equations
HSCL-25: Hopkins Symptom Checklist-25
HTQ: Harvard Trauma Questionnaire
ILO: International Labour Organization
IOM: International Office of Migration
IPV: Interpersonal violence
LK: Ligia Kiss
LSHTM: London School of Hygiene and Tropical Medicine
NET: Narrative exposure therapy
PRs: Prevalence ratios
PTSD: Post-traumatic stress disorder
QIC: Quasi-likelihood under the Independence Model Criterion
SD: Standard deviation
STEAM: Study on Trafficking, Exploitation and Abuse in the Mekong Sub-region
TF-CBT: Trauma-Focused Cognitive-Behavioral Therapy
WHO: World Health Organization
